# From immune activation to symptom burden: mechanisms and translational implications of immune–symptom uncoupling in Sjögren’s disease

**DOI:** 10.3389/fimmu.2026.1882936

**Published:** 2026-09-09

**Authors:** Jingying Le, Bingyan Gu, Zouyin Su, Xinchang Wang

**Affiliations:** 1 The Second Clinical Medical College of Zhejiang Chinese Medical University, Hangzhou, China; 2 The First School of Clinical Medicine, Zhejiang Chinese Medical University, Hangzhou, China; 3 Department of Rheumatology, The Second Affiliated Hospital of Zhejiang Chinese Medical University, Hangzhou, China

**Keywords:** biomarker–phenotype discordance, ESSDAI, ESSPRI, immune–symptom uncoupling, patient stratification, Sjögren’s disease, tissue–function discordance, treatment response

## Abstract

Sjögren’s disease (SjD) is a heterogeneous autoimmune disease in which systemic activity, local tissue pathology, objective glandular or ocular function, biomarkers, and patient-reported symptoms frequently fail to align or change in parallel. We define immune–symptom uncoupling as partial, non-linear, or temporally asynchronous discordance across these domains rather than complete biological separation. This focused narrative review was informed by targeted, structured searches of PubMed and the Web of Science Core Collection through July 9, 2026. We distinguish four recurrent forms: systemic activity–symptom, tissue–function, biomarker–phenotype, and response-layer discordance. The most consistent clinical evidence concerns weak alignment between systemic disease activity and dryness, pain, or fatigue. Multimodal and longitudinal studies further indicate that histopathology, ultrasonography, objective glandular function, and patient-reported dryness provide complementary rather than interchangeable information. Interferon activity, B-cell-associated markers, and autoantibody profiles may identify pathway activity, endotypes, or organ-risk biology without proportional symptom burden, while therapeutic studies show that biological response may occur without parallel systemic, tissue-level, functional, or symptomatic improvement. Tissue compartmentalization, active functional or bioenergetic dysregulation, structural injury and reduced functional reserve, SjD-related neurological involvement, central sensitization and symptom amplification, comorbid or contextual modifiers, and temporal asynchrony are plausible contributors; however, much of the mechanistic evidence remains observational or cross-sectional, and prospective causal validation is limited. Clinically, discordance should prompt targeted evaluation of local, functional, neurological, and contextual contributors rather than automatic escalation of immunosuppression or dismissal of symptoms. In trials, aligning the therapeutic target, enrolled population, primary endpoint, and assessment window with the expected response layer may reduce treatment-effect dilution and improve interpretation of negative or layer-specific findings. This framework supports more precise interpretation of disease activity, symptom burden, biomarkers, and treatment response while defining priorities for prospective multidomain studies.

## 1 Introduction

Sjögren’s disease (SjD) is a heterogeneous autoimmune disease with diverse clinical, biological, and patient-reported manifestations (1, 2). Across these domains, systemic immune activation, local tissue pathology, glandular dysfunction, and patient-reported symptoms often fail to align in a linear or synchronous manner. Dryness, pain, and fatigue correlate only weakly with systemic disease activity scores and, in many patients, show limited concordance with objective glandular measures (3–6). These observations suggest that symptom burden is not a direct readout of inflammatory intensity, but may reflect the combined influence of immune, tissue, functional, neural, and contextual processes that do not necessarily remain aligned during disease evolution.

This discordance has practical consequences. Patients with prominent dryness, pain, or fatigue may have little measurable systemic activity, whereas patients with serological, molecular, or organ-domain activity may report relatively modest symptoms. Residual symptoms after immune-targeted therapy may reflect glandular damage, reduced functional reserve, altered symptom processing, or delayed downstream recovery, even when there is evidence of biological response. These patterns challenge the assumption that SjD severity can be represented along a single axis from mild to severe disease.

In this review, we use immune–symptom uncoupling to describe non-linear, non-parallel, or temporally asynchronous discordance along the immune-to-symptom continuum. The concept refers to partial discordance rather than complete biological separation. For conceptual precision, we distinguish four recurrent forms: (1) systemic activity–symptom discordance, exemplified by weak alignment between systemic disease activity and patient-reported burden; (2) tissue–function discordance, in which histopathological, imaging, and functional measures of glandular or ocular involvement are not interchangeable; (3) biomarker–phenotype discordance, in which serological or molecular pathway activity does not translate into proportional clinical or symptomatic expression; and (4) response-layer discordance, in which biological, systemic, tissue-level, functional, and symptomatic responses do not occur in parallel. These forms may overlap, coexist, or change over time within the same patient.

Existing reviews have substantially advanced the understanding of SjD heterogeneity and precision medicine (1, 2), patient burden and outcome assessment (7–9), and evolving pathogenic mechanisms (10). These approaches are well suited to describing patient heterogeneity, molecular endotypes, and potential treatment stratification, but they do not directly organize a different problem: why valid measures from different disease domains may disagree within the same patient or diverge over time. We therefore use the relationship between domains, rather than the patient subgroup or candidate mechanism, as the unit of analysis. The four forms were selected because they represent recurrent observable interfaces at which inference across domains may fail: systemic activity versus symptom burden, tissue pathology versus objective function, biomarker activity versus clinical phenotype, and biological target engagement versus downstream clinical response. Candidate contributors are considered separately because the same process may contribute to more than one form of discordance. This organization spans baseline assessment through treatment response, allows multiple forms to coexist or change over time within the same patient, and clarifies what can—and cannot—be inferred from a given measure. Its added value is therefore complementary rather than competitive with heterogeneity and precision-medicine frameworks: it identifies where alignment fails and how that failure should affect clinical interpretation and trial design. We do not present immune–symptom uncoupling as a new biological mechanism or disease subtype, and the framework remains interpretive and hypothesis-generating rather than a validated classification system. Throughout this review, we use “Sjögren’s disease” (SjD), while retaining historical terminology when referring to original study populations and in the formal names of validated instruments, study cohorts, or article titles. The review proceeds from candidate contributors across systemic, tissue, functional, neural, and temporal domains to biomarker–phenotype discordance and recurrent clinical patterns, and finally to implications for immune-targeted trial design and future research.

## 2 Review approach and evidence interpretation

This focused narrative review was informed by targeted, structured searches of PubMed and the Web of Science Core Collection, covering database inception through July 9, 2026. Searches covered five domains: systemic disease activity and patient-reported symptoms; glandular and ocular pathology, imaging, and function; circulating biomarkers, immune pathways, and molecular or clinical phenotypes; neurological involvement and other contributors to symptom burden; and clinical trials, treatment response, endpoint behavior, and patient stratification. Reference lists of relevant reviews and key primary studies were also examined to identify additional publications.

We prioritized publications that directly informed relationships among these domains. Original studies were preferentially used to support specific empirical statements, whereas reviews, consensus documents, and instrument-development studies were used to establish broader context, terminology, and measurement principles. Evidence interpretation was guided by study design, sample size and population, directness of the comparison, consistency and replication across studies, and the availability of longitudinal or interventional support. Established clinical observations were distinguished from primarily associative findings, emerging mechanistic evidence, and hypothesis-generating interpretations. Representative study-level evidence and its principal limitations are summarized in **Table 1**. Consistent with the focused narrative scope, study selection emphasized relevance and evidentiary contribution rather than exhaustive literature coverage.

**Table 1:** Representative evidence for immune–symptom uncoupling in Sjögren’s disease.

Form of uncoupling	Representative study, design, and population	Domains/readouts compared	Key finding and evidence boundary
Systemic activity–symptom	Seror et al., 2015 (3); prospective 6-month ESSDAI/ESSPRI validation; n = 395, 15 countries.	Systemic vs patient-reported scores; responsiveness	Systemic–patient score correlations were very low (*r* = 0.07–0.29), while systemic scores were more responsive to improvement. Psychometric validation; mechanisms were not assessed.
	McCoy et al., 2022 (56); two-cohort cluster study; SICCA registry/survey cohort, n = 1,454/2,920.	Dryness, fatigue, and pain vs objective severity, HRQoL, and medication use	The high-symptom cluster had poorer HRQoL despite milder objective disease and more immunomodulator use. Cross-sectional and partly self-reported; medication patterns were descriptive.
Tissue–function	Mossel et al., 2022 (13); cross-sectional same-gland multimodal study; n = 41 meeting SjD criteria.	Parotid histopathology vs SGUS vs gland-specific stimulated salivary flow	Inflammatory histology showed moderate-to-good correlations with SGUS, whereas salivary flow was weakly related to either modality. Small cross-sectional sample; symptoms and temporality were not assessed.
	Sluijpers et al., 2023 (16); prospective standard-of-care cohort; n = 253 through year 2, of whom 75 had reached year 5.	SGUS and sialometry vs ESSPRI general dryness, oral dryness NRS, and Xerostomia Inventory	Baseline SGUS–PROM associations were poor and sialometry–PROM associations fair; objective changes did not correlate with PROM changes. Year-5 data were limited (n = 75); long-standing disease and observational design limit inference on early change or treatment effects.
	Mejía-Salgado et al., 2026 (18); retrospective comparative DED cohort; n = 324 (162 SjD/162 non-SjD DED).	OSDI vs ocular surface staining, NIBUT, and Schirmer test; sign–symptom discordance scores	SjD showed lower OSDI despite worse objective ocular findings, with sign-dominant discordance. Retrospective design; mechanisms and longitudinal sign–symptom trajectories were not assessed.
Biomarker–phenotype	Bodewes et al., 2018 (51); molecular discovery cohort (n = 50) and two independent European SjD cohorts (n = 86/55).	Systemic IFN-I/IFN-I+II activity vs ESSDAI domains, ESSPRI, and symptom components	IFN activity was associated with serological markers and the ESSDAI biological domain, but not total ESSDAI, ESSPRI, fatigue, or dryness; pain was lower in IFN-active groups. Predominantly cross-sectional; causality and treatment prediction were not assessed.
	Nguyen et al., 2024 (1); Nguyen et al., 2025 (53); cluster derivation (n = 534) and validation/biomarker analysis in the prospective ASSESS cohort (n = 395).	Symptoms, ESSDAI, and routine laboratory measures vs CXCL13, IL-7, TNF-RII, and IFN signature	Three clusters were reproduced; CXCL13, IL-7, and TNF-RII were higher in BALS/HSA than LSAHS, while high IFN signature was most frequent in BALS. Observational clustering; biomarker/prognostic analyses were confined to ASSESS, with sparse events and wide confidence intervals.
	Fonzetti et al., 2025 (60); n = 279 anti-SSA/Ro-positive patients meeting SjD classification criteria.	Anti-Ro52, anti-Ro60, and anti-La/SSB profiles vs ESSDAI, IgG, ESSPRI, pain, and fatigue	Triple-positive patients showed the highest ESSDAI and IgG levels, whereas isolated anti-Ro60 patients had a median ESSDAI of 0 yet the highest ESSPRI. Seropositive-only, single-center, and cross-sectional; external and longitudinal validation is lacking.
Response-layer	Baer et al., 2021 (78); phase III randomized, double-blind, placebo-controlled trial; n = 187 anti-SSA/Ro-positive patients with active SjD.	Abatacept effects on IgG, IgA, IgM-RF, and immune-cell subsets vs ESSDAI, ESSPRI, and stimulated salivary flow	Abatacept altered disease-relevant biomarkers and pathogenic cell subsets but did not improve ESSDAI, ESSPRI, or stimulated salivary flow vs placebo. Biomarker/cell analyses were partly *post hoc*; restrictive eligibility and substantial placebo improvement in ESSDAI limit interpretation.
	Bowman et al., 2017 (79); Fisher et al., 2018 (80); multicenter rituximab RCT/SGUS substudy; n = 133/52.	Symptom primary endpoint, ESSPRI, and ESSDAI vs unstimulated flow and SGUS	The symptom endpoint was negative; rituximab preserved unstimulated flow and improved the total SGUS score vs placebo. Flow was a secondary outcome; the small SGUS substudy used a novel score, with multiplicity concerns.
	Collins et al., 2021 (91); *post hoc* stratified reanalysis of a hydroxychloroquine trial; n = 107 (IFN data, n = 68).	Hydroxychloroquine effects on ESSPRI vs type I IFN score	ESSPRI differences favored HCQ in HSB/LSB without IFN reduction, whereas IFN scores fell in PDF/DDF without ESSPRI benefit. *Post hoc* and underpowered for subgroup effects; IFN data were unavailable at week 12.

Studies were selected as representative rather than exhaustive examples. Study design and population are reported explicitly to indicate the nature and directness of the supporting evidence, while evidence boundaries summarize design-specific limitations. Slash-separated sample sizes correspond to the listed cohorts/analyses in order. ASSESS, Assessment of Systemic Signs and Evolution of Sjögren’s Syndrome; BALS, B-cell active with low symptoms; CXCL13, C-X-C motif chemokine ligand 13; DDF, dryness dominant with fatigue; DED, dry eye disease; ESSDAI, EULAR Sjögren’s Syndrome Disease Activity Index; ESSPRI, EULAR Sjögren’s Syndrome Patient Reported Index; HCQ, hydroxychloroquine; HRQoL, health-related quality of life; HSA, high systemic activity; HSB, high symptom burden; IFN, interferon; IgA, immunoglobulin A; IgG, immunoglobulin G; IgM-RF, immunoglobulin M rheumatoid factor; IL-7, interleukin-7; LSAHS, low systemic activity with high symptom burden; LSB, low symptom burden; NIBUT, non-invasive tear break-up time; NRS, numeric rating scale; OSDI, Ocular Surface Disease Index; PDF, pain dominant with fatigue; PROM, patient-reported outcome measure; RCT, randomized controlled trial; SGUS, salivary gland ultrasonography; SICCA, Sjögren’s International Collaborative Clinical Alliance; SjD, Sjögren’s disease; TNF-RII, tumor necrosis factor receptor II.

## 3 Candidate biological and clinical contributors to immune–symptom uncoupling

**Figure 1:**
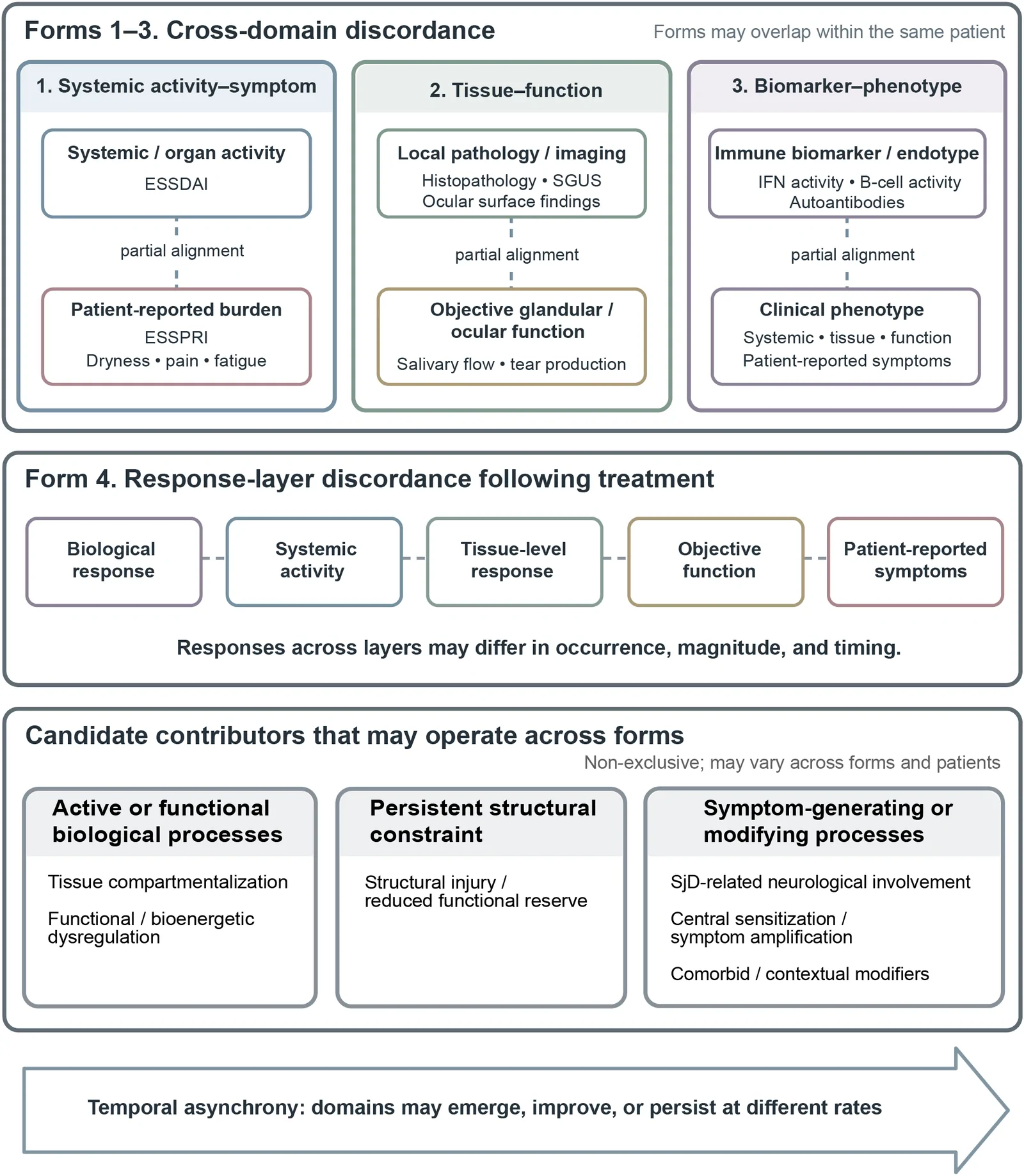
Conceptual framework for four recurrent forms of immune–symptom uncoupling in Sjögren’s disease. Forms 1–3 depict cross-domain discordance: (1) systemic activity–symptom discordance, in which systemic or organ activity may align only partially with patient-reported burden; (2) tissue–function discordance, in which local histopathological or imaging abnormalities may not parallel objective glandular or ocular function; and (3) biomarker–phenotype discordance, in which immune biomarkers or endotypes may not translate into proportional systemic, tissue, functional, or symptomatic expression. These cross-domain patterns may be observed cross-sectionally or evolve longitudinally. Form 4 depicts response-layer discordance after treatment, in which biological, systemic, tissue-level, functional, and patient-reported responses may differ in occurrence, magnitude, and timing. Biological response encompasses target engagement or changes in pathway-related biomarkers, whereas tissue-level response encompasses histopathological or imaging change. The left-to-right arrangement represents distinct response layers rather than an obligatory biological sequence, and dashed connectors indicate partial or non-parallel alignment rather than deterministic causation. Candidate contributors are grouped conceptually as active or functional biological processes (tissue compartmentalization and functional/bioenergetic dysregulation), persistent structural constraint (structural injury and reduced functional reserve), and symptom-generating or modifying processes (SjD-related neurological involvement, central sensitization or symptom amplification, and comorbid or contextual modifiers). These contributors are non-exclusive, their relative importance may differ across forms and individual patients, and no one-to-one mapping between a contributor and a specific form is implied. Temporal asynchrony may affect all four forms. ESSDAI, EULAR Sjögren’s Syndrome Disease Activity Index; ESSPRI, EULAR Sjögren’s Syndrome Patient Reported Index; IFN, interferon; SGUS, salivary gland ultrasonography; SjD, Sjögren’s disease.

The four forms defined above are overlapping patterns rather than distinct mechanisms. **Figure 1** summarizes selected candidate contributors that may operate across forms without implying that all are present in every patient or form a fixed causal sequence.

Systemic activity–symptom discordance is the form most consistently documented in clinical studies. The EULAR Sjögren’s Syndrome Disease Activity Index (ESSDAI) and EULAR Sjögren’s Syndrome Patient Reported Index (ESSPRI), which assess systemic organ-domain activity and patient-reported dryness, pain, and fatigue, respectively, correlate only weakly (3, 11, 12). Because these instruments were intentionally developed to measure different constructs, weak correlation is partly expected and, by itself, should not be interpreted as evidence of a specific biological mechanism underlying uncoupling. This supports their non-interchangeability at the construct level but does not identify the source of discordance in an individual patient. Biological support for uncoupling is stronger when multimodal or longitudinal data demonstrate non-parallel relationships among immune activity, tissue pathology, objective function, biomarkers, and patient-reported symptoms within the same patients.

### 3.1 Partial alignment across glandular and ocular pathology, function, and symptoms

Histopathology, imaging, secretory function, and patient-reported symptoms capture distinct aspects of salivary, lacrimal, and ocular-surface involvement. In same-gland comparisons, histopathological inflammation correlated more closely with salivary gland ultrasonography (SGUS) abnormalities than with stimulated salivary flow, which showed limited alignment with either modality (13). In the large Sjögren’s International Collaborative Clinical Alliance (SICCA) registry, a focus score of ≥1 was strongly associated with anti-SSA/Ro and/or anti-La/SSB positivity and objective ocular abnormalities, but not with patient-reported dry-eye or dry-mouth symptoms (14).

Subjective and objective oral and ocular dryness show substantial interindividual variation, although associations with lacrimal or salivary flow differ by instrument and the aspect of dryness assessed (4, 5, 15). In a prospective cohort, SGUS findings, salivary flow, and dryness-related patient-reported outcomes remained broadly stable at the group level over follow-up, whereas changes in objective glandular assessments did not closely correlate with symptom changes (16). At the ocular surface, SjD-specific data provide more direct evidence of sign–symptom discordance. Large SICCA analyses showed substantially worse objective ocular abnormalities in SjD despite similar symptom burden compared with non-SjD participants (17). A more recent SjD-versus-non-SjD dry-eye cohort similarly found lower Ocular Surface Disease Index (OSDI) scores despite more severe objective findings, with formal discordance analyses indicating greater sign than symptom severity in SjD (18). Corneal sensory and nerve abnormalities may further modify symptom perception (19); neuropathic ocular pain has also been described (20), while coexisting meibomian gland dysfunction may add an evaporative component (21). Longitudinal data from the Dry Eye Assessment and Management (DREAM) further suggest that ocular signs and symptoms may follow non-parallel trajectories, although these observations were derived from a broader dry-eye population rather than an SjD-specific cohort (22). Direct longitudinal evidence linking these specific mechanisms to sign–symptom discordance in SjD remains limited.

Cellular and spatial studies provide biological context rather than direct proof of the mechanisms. Salivary gland epithelial cells can participate in antigen presentation, cytokine and chemokine production, B-cell activation, and local inflammatory amplification (23). Single-cell and spatial analyses have also identified organized immune, stromal, and vascular-associated cell states within glandular tertiary lymphoid structures (24). Overall, direct multimodal comparisons and longitudinal observations support incomplete tissue–function and tissue–symptom alignment. Causal mechanisms and temporal ordering remain uncertain because most comparisons are observational, tissue cohorts are small, and longitudinal data largely concern established disease.

### 3.2 Active functional dysregulation may occur without fixed structural loss

Persistent dysfunction may also reflect biologically active disturbances of cellular or receptor-level function that are not synonymous with irreversible tissue loss. Muscarinic cholinergic signaling provides a candidate example in exocrine glands. In an early functional study, immunoglobulin G (IgG) from patients with SjD acutely and reversibly inhibited carbachol-evoked calcium signaling and downstream ion-channel activation in human and mouse submandibular acinar cells; direct intracellular activation with inositol 1,4,5-trisphosphate bypassed this inhibition, supporting a receptor-proximal mechanism (25). Subsequent work, however, highlighted substantial cross-reactivity among muscarinic receptor targets and limitations of peptide- and transfectant-based binding assays in distinguishing pathogenic from naturally occurring antibodies (26). More recently, autoantibodies against multiple muscarinic receptor subtypes were detected at increased frequencies in a large SjD cohort, and purified SjD IgG reduced calcium signaling in M1R-, M3R-, and M5R-expressing cells (27). These findings support receptor-level interference as a plausible, potentially non-destructive contributor to glandular dysfunction. Nevertheless, antibody detection does not establish functional activity, and muscarinic autoantibody positivity was not associated with objective oral or ocular dryness measured by Saxon or Schirmer testing.

A related form of active functional dysregulation may contribute to fatigue. In a small case–control study, circulating T cells from patients with SjD showed reduced basal and ATP-linked mitochondrial respiration, maximal respiration, and reserve capacity compared with healthy controls, while selected discomfort- and sleep-related measures correlated with mitochondrial respiration; transcriptomic analyses also identified heterogeneous mitophagy-related programs (28). However, similar respiratory abnormalities were observed in non-Sjögren sicca, the functional cohort was small, and associations between mitophagy-defined subgroups and fatigue were only suggestive. Mitochondrial bioenergetic dysregulation should therefore be regarded as an emerging mechanistic hypothesis rather than an established or SjD-specific explanation for fatigue.

### 3.3 Structural injury and reduced functional reserve may sustain dysfunction

In SjD, current immune activity and current glandular function are not always synchronized because prior inflammation may reduce epithelial integrity, alter ductal architecture, promote fibrosis, deplete secretory reserve, or impair glandular homeostasis. When such changes are established, dryness and secretory dysfunction may persist even when systemic inflammatory activity has declined or remains limited.

ESSDAI was developed to assess current organ-domain activity and does not score fixed sequelae as active disease (12). A low systemic activity score may therefore coexist with persistent glandular dysfunction associated with accumulated structural injury, reduced functional reserve, or chronic epithelial dysfunction.

Minor salivary gland fibrosis is increased in SjD and is associated with focus score, indicating that fibrosis is linked to the disease process rather than representing only age-related background change (29). Stimulated salivary flow has also been associated with biopsy-based focus score and fibrosis, suggesting that glandular output may reflect both inflammatory and damage-related components of glandular pathology (30). Recent reviews further emphasize that salivary gland fibrosis involves immune and non-immune mediators and remains an under-investigated potential contributor to chronic glandular dysfunction (31).

In a cohort of 51 anti-SSA/Ro-positive patients with SjD, greater glandular preservation was associated with better baseline salivary function, whereas higher ductal epithelial p16 expression independently predicted subsequent decline in unstimulated salivary flow (32). These findings suggest that structural integrity and epithelial senescence may help define residual functional capacity, although validation in larger and more diverse cohorts is required. Experimental studies outside SjD suggest that p16 can be functionally linked to the senescence-associated secretory phenotype (SASP), although p16 expression alone is not sufficient to define SASP activity. p16 suppression reduces multiple SASP factors, while chronic p16-associated senescence in corneal endothelial cells is accompanied by a cytokine-rich secretome with paracrine inflammatory and profibrotic effects (33, 34). If present in SjD, such paracrine signaling could provide a route from epithelial senescence to persistent local dysfunction without proportional lymphocytic infiltration; this mechanism has not been demonstrated directly in SjD salivary tissue.

Evidence linking structural pathology to glandular dysfunction is therefore supported mainly by cross-sectional biopsy–function associations, with emerging longitudinal evidence relating epithelial senescence to subsequent salivary decline. However, direct relationships with patient-reported dryness, reversibility of structural abnormalities, and functional or symptomatic recovery after immune modulation remain insufficiently defined. Structural injury and reduced functional reserve should therefore be regarded as plausible contributors to persistent dysfunction rather than universal explanations for residual symptoms.

### 3.4 Neurological, symptom-amplifying, and contextual contributors to symptom burden

Neurological involvement, central sensitization, and comorbid or contextual modifiers may all contribute to symptom burden in SjD, but they represent distinct clinical and mechanistic domains.

#### 3.4.1 SjD-related neurological involvement

Peripheral nervous system involvement is a recognized extraglandular manifestation of SjD and includes distal axonal polyneuropathy, sensory neuronopathy, mononeuropathies, small-fiber neuropathy, and autonomic neuropathy (35, 36). A systematic review and meta-analysis of 49 studies estimated a pooled prevalence of peripheral neuropathy of approximately 15%, although estimates varied according to study population, neuropathy definition, and ascertainment method (35). These manifestations may cause pain, sensory disturbance, ataxia, autonomic complaints, and functional limitation that are not captured by glandular measures.

Small-fiber neuropathy is particularly relevant because burning pain, dysesthesia, allodynia, and autonomic symptoms may occur despite normal conventional nerve conduction studies. In a prospective series of 40 patients with SjD-associated small-fiber neuropathy, diagnosis was supported by small-fiber neurophysiological abnormalities or reduced intraepidermal nerve-fiber density, illustrating the need for investigations beyond standard large-fiber testing (37). Neuropathic sensory features may also affect ocular, oral, and bodily symptom perception, although direct evidence linking these features to ocular sign–symptom discordance remains limited (20).

Autonomic involvement represents a related but distinct source of burden. In a prospective cohort of 154 patients, altered autonomic activity was associated with greater fatigue but not with objective glandular manifestations (38). In a later cohort of 266 patients, autonomic symptoms showed limited relationships with objective glandular or systemic measures but were associated with pain, fatigue, dryness, high-symptom phenotypes, and work disability (39). The latter study assessed patient-reported autonomic symptoms rather than objectively confirmed autonomic neuropathy.

Evidence strongly supports the clinical diversity of SjD-related neurological involvement, but its specific contribution to immune–symptom uncoupling remains less certain because diagnostic methods are heterogeneous and longitudinal symptom-linked studies are scarce.

#### 3.4.2 Central sensitization and symptom amplification

Central sensitization refers to altered central processing of sensory input and is distinct from peripheral neuropathy, active systemic inflammation, and structural glandular damage. In SjD, higher central sensitization scores have been associated with poorer sleep quality, greater pain and fatigue, worse functional status, and higher patient-reported burden (40, 41). Such amplification does not imply that symptoms are psychologically generated or clinically insignificant.

Current evidence is limited mainly to small, cross-sectional studies using questionnaire-based measures. These studies support associations with symptom burden and disability but do not establish temporal direction, SjD specificity, or treatment responsiveness. Central sensitization should therefore be regarded as a potential symptom-amplifying contributor rather than a confirmed mechanism in an individual patient.

#### 3.4.3 Comorbid and contextual modifiers

Comorbid and contextual factors may modify symptom burden without necessarily reflecting greater systemic immune activity. Fibromyalgia provides the clearest example. In a recent multicenter cohort of 267 patients with SjD, concomitant fibromyalgia was associated with higher ESSPRI scores and greater pain, fatigue, and dryness, whereas ESSDAI scores were comparable between patients with and without fibromyalgia (42). Earlier cohort data similarly identified greater patient-reported burden without proportionally greater systemic activity, although prevalence estimates varied according to the criteria used (43). Fibromyalgia should therefore be recognized as a relevant comorbidity without being used to dismiss symptoms or exclude coexisting active SjD.

Sleep, mood, and cognitive–affective factors may also influence symptom expression. In a multicenter cohort of 608 patients, pain, depressive symptoms, and daytime sleepiness were independently associated with both physical and mental fatigue after adjustment for relevant comorbidities and medications (44). Pain catastrophizing and negative illness perceptions have likewise been associated with greater pain severity, although the direction of these relationships remains uncertain (45).

Medication effects and non-SjD conditions should also be considered. Anticholinergic and other xerogenic medications may aggravate dryness, while sedating medications may contribute to fatigue. In a population-based study of 4,945 patients with SjD, 39.1% received at least one medication with anticholinergic properties (46). Anemia, thyroid disease, diabetes, primary sleep disorders, and other medical conditions may also contribute to symptom burden.

Evidence for these modifiers is predominantly observational and does not establish causality or SjD specificity. Their identification should prompt diagnostic separation without assuming that SjD-related inflammation, neurological involvement, or local glandular and ocular disease is absent.

### 3.5 Temporal asynchrony contributes to non-parallel trajectories

Systemic immune activity, tissue-compartment pathology, glandular function, structural damage, biomarkers, and patient-reported symptoms may emerge, improve, or persist at different rates. A single visit may therefore capture these domains at different points in their trajectories, making dynamic divergence appear as a stable discordance between “objective” and “subjective” disease.

In prospective cohorts, objective glandular assessments and patient-reported measures may remain broadly stable at the group level, while changes in objective glandular parameters do not closely parallel changes in symptoms within individuals (16). Longer time spent in ESSDAI-defined low disease activity has been associated with better salivary gland outcomes (47). In a large national registry, systemic activity decreased between inclusion and follow-up, whereas patient-reported dryness, pain, and fatigue increased over the same period (48).

In rituximab-treated patients with SjD, ESSDAI and ESSPRI may both show responsiveness, but ESSDAI showed greater responsiveness, while changes in stimulated salivary flow remained small over the same assessment period (49). These findings are consistent with domain-specific response patterns, but cannot determine whether the differences reflect response kinetics, limited reversibility, or the properties of the measurement instruments.

Longitudinal cohort and repeated-measures treatment studies therefore support the presence of non-parallel trajectories in SjD. However, few studies have repeatedly assessed immune biomarkers, tissue pathology, objective function, and symptoms within the same patients, and assessment intervals may not correspond to the kinetics of each domain. Temporal asynchrony remains a plausible explanation for some observed discordance, but distinguishing delayed recovery from structural persistence or stable symptom-modifying factors requires prospective multidomain follow-up.

## 4 Biomarker–phenotype discordance across immune, tissue, and symptom domains

Biomarker–phenotype discordance occurs when a serological or molecular readout indexes biological activity without proportional clinical or symptomatic expression. Discordance should therefore be used to identify the domain being measured rather than interpreted as failure of either the biomarker or the symptom measure.

### 4.1 Circulating immune biomarkers and immune pathway activity

Circulating markers associated with B-cell and systemic immune activity include serum β2-microglobulin and immunoglobulin free light chains; higher levels of these markers have been associated with greater systemic disease activity, but they have not been established as direct measures of patient-reported symptom burden (50). Interferon (IFN) activation is itself heterogeneous: patients may be IFN-inactive, show predominant type I IFN activity, or combined type I and type II IFN activity (51). These IFN-defined states differed in biological activity, whereas ESSPRI, fatigue, and dryness did not differ significantly between groups; pain scores were lower in the IFN-active groups. Thus, B-cell-associated and IFN biomarkers may identify biologically distinct immune states without proportional symptom expression. Multi-omic classification studies extend this point beyond single biomarkers. Such molecular classes are best viewed as immune endotypes: they may inform pathway activity, biological stratification, and hypotheses for pathway-targeted treatment selection, but they do not by themselves define symptom burden (52). A prospective biomarker analysis of the Assessment of Systemic Signs and Evolution of Sjögren’s Syndrome (ASSESS) cohort found that inflammatory and B-cell-associated markers were higher in B-cell-active/low-symptom and high-systemic-activity subgroups than in the low-systemic-activity/high-symptom subgroup (53). These classification approaches remain limited by differences in study populations, biological measurements, and analytical methods, as well as by limited external and longitudinal validation. More recent studies have extended circulating biomarker assessment to blood-count-derived inflammatory indices and multiplex serum profiling. In a retrospective multicenter cohort (n = 239), the neutrophil-to-lymphocyte ratio and systemic immune-inflammation index were higher in extraglandular SjD with moderate-to-high ESSDAI, whereas no corresponding differences were observed with high ESSPRI (54). In a smaller cross-sectional study, multiplex profiling of 23 serum mediators identified five that differed between SjD and controls; among them, monocyte chemoattractant protein-1 showed modest correlations with focus score and sicca severity (55). Thus, evidence is strongest for associations of circulating biomarkers with systemic activity, laboratory abnormalities, and selected clinical phenotypes; longitudinal relationships with symptom trajectories and treatment response remain insufficiently defined.

### 4.2 Symptom-defined phenotypes capture burden architecture

Symptom-defined phenotypes provide a complementary map of SjD heterogeneity by organizing the patient-experienced burden itself. Dryness, pain, fatigue, anxiety, and depression can cluster into reproducible patient subgroups, revealing structured patterns of symptom expression across cohorts (6, 56–58).

In the international stratification study by Tarn et al., patients with SjD were grouped according to pain, fatigue, dryness, anxiety, and depression, generating symptom-based subgroups with distinct clinical and biological associations (6). McCoy et al. similarly identified symptom-based SjD clusters across international cohorts and highlighted discordance between symptom burden, end-organ manifestations, and treatment patterns (56). Longitudinal work further suggests that symptom-defined subgroups may show persistence over time and differ in clinical trajectories (57). More recent Newcastle Sjögren’s Stratification Tool (NSST)–based analyses also support the stability of symptom-based subtype membership in a large proportion of patients, reinforcing the view that symptom phenotypes represent structured patterns of disease expression (58). However, subgroup definitions have varied according to the symptoms included, analytical methods, and study populations, and their longitudinal stability has not been established uniformly across cohorts.

Symptom-defined and immune-defined phenotypes therefore provide complementary, potentially overlapping descriptions of SjD heterogeneity. Symptom-based stratification may help identify clinically relevant patterns of patient-experienced burden, whereas immune phenotypes may be more informative for pathway activity and organ-risk assessment. However, the use of either classification for treatment selection or trial enrichment requires prospective validation of subgroup stability and differential treatment response.

### 4.3 Autoantibody-defined subsets point to immune and tissue programs

Autoantibody-defined subsets provide a clinically familiar example of biomarker interpretation in SjD.

Anti-SSA/Ro-positive disease is often linked to IFN-related biological activity (51), whereas symptom expression may vary across patients with the same broad serological label. More refined antigenic profiling suggests that anti-Ro52, anti-Ro60, and anti-La/SSB combinations may index different immune-program structures rather than a single serological severity gradient. In particular, combined anti-Ro52/TRIM21 and anti-Ro60/SSA positivity has been associated with greater systemic disease severity and stronger IFN pathway activation (59).

In a real-life cohort, Fonzetti et al. reported that distinct anti-Ro52, anti-Ro60, and anti-La/SSB profiles were associated with different systemic and patient-reported patterns. Triple positivity for anti-Ro52, anti-Ro60, and anti-La/SSB was associated with the highest systemic inflammatory activity, higher IgG levels, and broader hematologic, lymphoid, glandular, and biological involvement. In contrast, isolated anti-Ro60 patients showed the lowest systemic activity and the highest frequency of fibromyalgia, while isolated anti-Ro52 and isolated anti-Ro60 subsets showed higher adjusted ESSPRI, pain, and fatigue scores. Low-activity/high-symptom cases were enriched in isolated anti-Ro52 and anti-Ro60 groups (60). These findings suggest that antigenic specificity may distinguish immune-active and symptom-dominant configurations. However, they derive from a single observational cohort and require replication in independent and longitudinal populations.

Tissue-compartment profiling links these serological subsets to local glandular programs. Comparative single-cell and spatial analyses of anti-SSA/Ro-positive and anti-centromere-positive SjD have shown both shared and distinct salivary gland programs, including IFN-associated immune activation in anti-SSA/Ro-positive disease and fibroblast-mediated inflammatory features in anti-centromere-positive disease (61). These findings suggest that autoantibody-defined subsets may correspond not only to circulating immune differences but also to distinct glandular microenvironments.

Beyond systemic or overlap features, anti-centromere antibody (ACA) positivity has been linked to glandular imaging abnormalities. In an SGUS study, ACA positivity was independently associated with SGUS score in patients with SjD, and ACA-positive patients showed higher ultrasound scores and more frequent hyperechoic bands in the major salivary glands (62). Together, these studies support associations between autoantibody-defined subsets and glandular molecular or imaging phenotypes. However, the evidence is predominantly cross-sectional and does not establish that these tissue features mediate specific symptoms or predict longitudinal functional and treatment responses.

Anti-aquaporin-5 (AQP5) antibodies represent a less established candidate autoantibody subset. In a small pilot study of 36 patients with SjD and eight healthy donors, overall anti-AQP5 levels did not differ between groups, but higher levels were associated with high systemic disease activity and elevated ESSPRI (63). Prior human and experimental evidence has linked anti-AQP5 reactivity to altered AQP5 function, glandular histopathology, and reduced salivary flow (64). Recent mouse T-cell experiments further demonstrated cross-recognition between AQP5 and aquaporin-4 epitopes, supporting AQP5 as a candidate autoantigen, although its relevance to SjD glandular autoimmunity and the causal role of circulating anti-AQP5 antibodies in human disease remain unproven (65).

Overall, evidence is strongest for associations between autoantibody profiles and systemic, laboratory, organ-associated, or tissue phenotypes. Associations with patient-reported burden are emerging but remain based mainly on observational cohorts, while longitudinal and treatment-predictive validation is limited. Most autoantibody profiles should therefore be interpreted as domain-specific stratification markers rather than global measures of disease or symptom severity. Functional autoantibodies directed against secretory receptors represent a conceptually distinct possibility because they may directly perturb receptor signaling; however, their assay reproducibility, clinical correlates, and treatment-predictive relevance remain incompletely established.

## 5 Clinical interpretation of recurrent discordance patterns

Clinically, discordance should prompt identification of the dominant modifiable process rather than automatic escalation of immunosuppression or dismissal of symptoms. Three recurrent presentations are considered below: symptom-high/activity-low disease, biomarker-high/symptom-modest disease, and persistent dysfunction after partial immune improvement. They may overlap or change over time and are summarized in **Table 2**.

**Table 2:** Assessment considerations and interpretive principles for recurrent clinical presentations in Sjögren’s disease.

Clinical presentation	Assessment considerations	Interpretive principle
Symptom-high/activity-low	Glandular and ocular domains: selected assessment guided by the presenting phenotype, including SGUS; unstimulated and stimulated salivary flow; ocular staining score, Schirmer testing, and tear-film stability; and evaluation for meibomian gland dysfunction or neuropathic ocular pain. Neurological involvement: evaluation for peripheral neuropathy, small-fiber neuropathy, or autonomic dysfunction when indicated. Symptom amplification and other modifiers: assess central sensitization or fibromyalgia when indicated; review xerogenic or sedating medications, sleep or mood disturbance, and other relevant comorbidities.	Low systemic activity does not exclude clinically relevant local or neurological involvement, but symptom burden alone does not establish ongoing targetable inflammation. Identify the dominant modifiable contributor before escalating immunosuppression.
Biomarker-high/symptom-modest	Systemic and organ domains: ESSDAI plus clinical, laboratory, or imaging assessment directed by the identified abnormality, suspected organ involvement, or risk domain. Hematologic and renal risk: selected testing guided by the identified abnormality, such as blood count, complement or cryoglobulins, or renal studies. Glandular and lymphoproliferative risk: assessment of persistent salivary gland swelling and targeted imaging or biopsy when indicated.	Modest symptom burden does not imply biologically mild disease. Interpret each abnormality within the biological, organ-specific, or prognostic domain it represents; biomarker positivity alone should neither mandate treatment escalation nor be assumed to predict treatment response.
Persistent dysfunction after partial immune improvement	Response layers: compare baseline and follow-up measures of target engagement, systemic activity, tissue or imaging findings, objective glandular or ocular function, and patient-reported symptoms separately. Residual burden: reassess structural damage and functional reserve, neurological involvement, central sensitization or fibromyalgia, medication effects, sleep disturbance, and other comorbid contributors when indicated. Timing: interpret each layer according to its expected response kinetics.	Interpret biological, systemic, tissue-level, functional, and symptomatic responses separately. Persistent dysfunction alone cannot distinguish incomplete immune control from structural damage, delayed recovery, or downstream symptom generation.

These clinical presentations may overlap and do not map one-to-one onto the four forms of uncoupling defined in this review. The assessments are illustrative rather than a minimum test set and do not constitute a validated clinical pathway; selection should be guided by the presenting phenotype, specific abnormality, suspected risk domain, and clinical context. ESSDAI, EULAR Sjögren’s Syndrome Disease Activity Index; SGUS, salivary gland ultrasonography.

### 5.1 Symptom-high/activity-low disease: localizing the symptom-generating process

**Figure 2:**
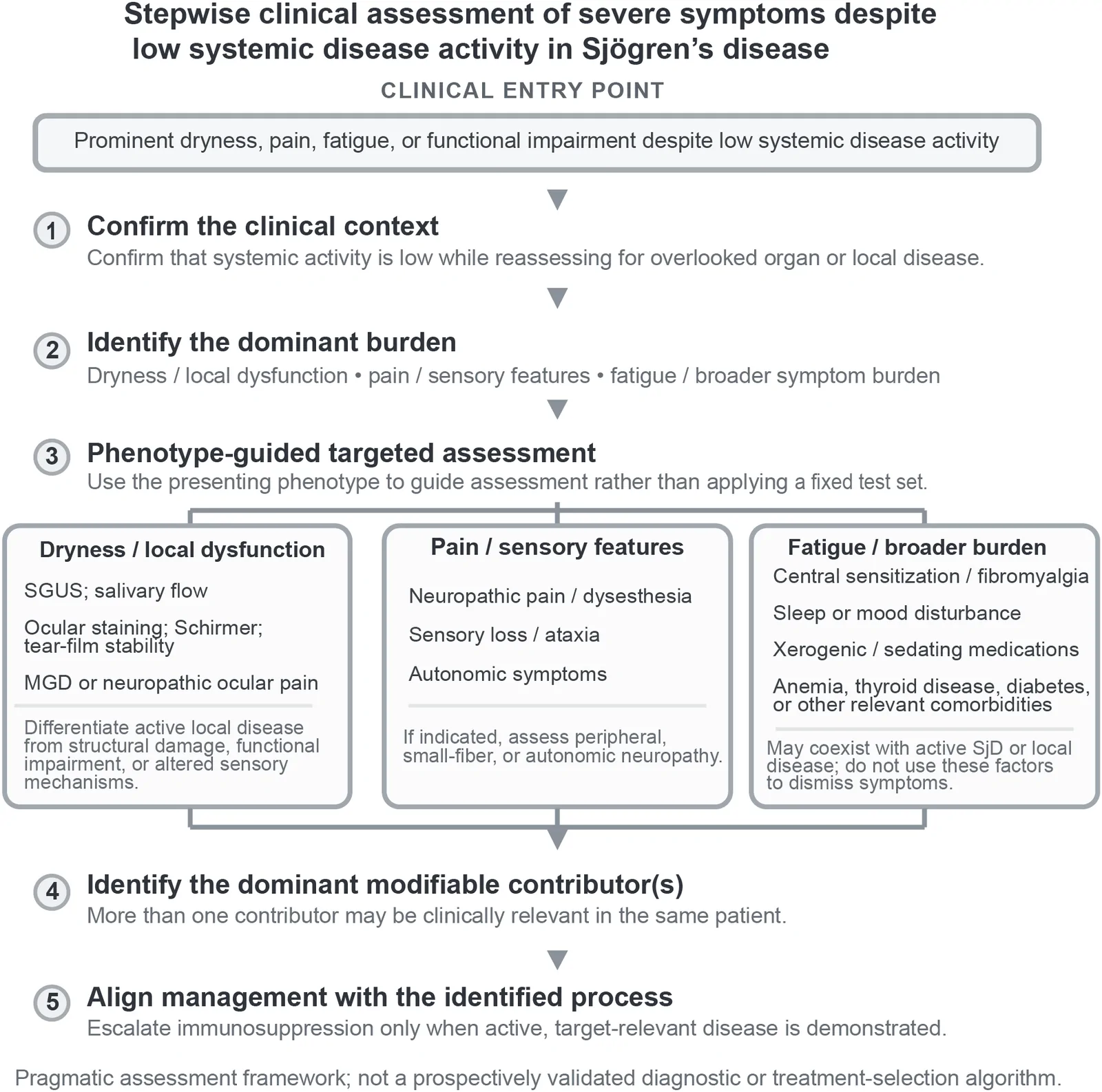
Stepwise clinical assessment of severe symptoms despite low systemic disease activity in Sjögren’s disease. The framework begins with patients presenting with prominent dryness, pain, fatigue, or functional impairment despite low systemic disease activity. Step 1 confirms the clinical context while reassessing for overlooked clinically relevant organ or local disease. Step 2 identifies the dominant burden, and Step 3 uses the presenting phenotype to guide targeted assessment across three non-exclusive domains: dryness or local dysfunction, pain or sensory features, and fatigue or broader symptom burden. Selected glandular, ocular, neurological, symptom-amplifying, medication-related, and comorbid contributors are then interpreted according to the presenting phenotype. Step 4 integrates these findings to identify the dominant modifiable contributor or contributors, recognizing that more than one process may be clinically relevant in the same patient. Step 5 aligns management with the identified process; escalation of immunosuppression should be considered only when active, target-relevant disease is demonstrated. The arrows indicate a pragmatic assessment sequence rather than a mandatory or prospectively validated diagnostic or treatment-selection algorithm. MGD, meibomian gland dysfunction; SGUS, salivary gland ultrasonography; SjD, Sjögren’s disease.

In clinical practice, immune–symptom uncoupling is often encountered as prominent dryness, pain, fatigue, or functional impairment despite limited measurable systemic activity (3, 6). This symptom-high/activity-low pattern includes several biologically distinct scenarios. The more useful question is which process is generating the burden: structural or functional persistence, compartmentalized glandular or ocular disease, SjD-related neurological involvement, central sensitization, comorbid or contextual modifiers, or a combination of these processes. A pragmatic stepwise assessment framework for this symptom-high/activity-low presentation is summarized in **Figure 2**.

One possibility is damage- or function-dominant persistence. Persistent sicca symptoms or reduced glandular output may be maintained by structural injury, reduced secretory reserve, or chronic epithelial dysfunction rather than by ongoing systemic immune escalation alone (12, 29–31). Here, low systemic activity may coexist with substantial disease burden when the dominant process has shifted downstream from active inflammation to structural or functional limitation. In this setting, escalation of immunosuppression for persistent dryness alone should be approached cautiously unless there is evidence of active, target-relevant inflammation.

A second possibility is glandular or ocular disease that is incompletely represented by systemic activity measures. SGUS, histopathology, salivary flow, tear-film assessments, and ocular surface staining capture different inflammatory, structural, functional, or surface-damage domains and should not be treated as interchangeable readouts (13, 15, 66–69). Abnormal local findings therefore require interpretation according to the process measured: evidence of current inflammation should be distinguished from structural abnormality, impaired function, and altered sensory or neuropathic pain mechanisms. In this situation, low systemic activity does not exclude clinically relevant local disease, but neither does every local abnormality establish ongoing targetable inflammation.

A third possibility includes neurological, symptom-amplifying, and comorbid or contextual contributors, which should be assessed separately rather than combined into a single neuroimmune category. Neuropathic pain, sensory loss, ataxia, autonomic complaints, or otherwise unexplained functional impairment may prompt evaluation for SjD-related peripheral or autonomic nervous system involvement (35–39). Widespread pain, severe fatigue, sleep disturbance, or disproportionate functional impact may instead indicate central sensitization or concomitant fibromyalgia (40–43). Mood symptoms and daytime sleepiness may further contribute to fatigue (44), while pain catastrophizing and illness perceptions may influence pain expression (45). Xerogenic or sedating medications should also be reviewed as potentially modifiable contributors (46). Anemia, thyroid disease, diabetes, and primary sleep disorders should be considered as alternative or coexisting causes. These processes may coexist with active SjD or local glandular and ocular disease and should not be used to dismiss patient-reported symptoms.

Depending on the dominant contributor, management may require coordinated input from rheumatology, ophthalmology, oral medicine or dentistry, neurology, pain or sleep medicine, and rehabilitation. These distinctions can guide targeted referral and multidisciplinary care, but they do not constitute a prospectively validated diagnostic or treatment-selection algorithm.

### 5.2 Biomarker-high/symptom-modest disease: assessing the risk layer

Biomarker-high/symptom-modest disease represents the reverse form of immune–symptom uncoupling. Serological, molecular, laboratory, imaging, or organ-domain activity may be present despite relatively modest dryness, pain, fatigue, or functional impact. A key interpretive error is to equate modest symptom burden with biologically mild disease.

A patient with a modest ESSPRI score may still require assessment of systemic domains, hematologic abnormalities, serological activity, imaging findings, and organ-specific risk. Systemic involvement remains central to risk assessment and therapeutic management in SjD, particularly when organ-domain disease requires systemic therapy rather than symptomatic treatment alone (70). Where indicated, evaluation should use organ-specific investigations rather than relying on global symptom burden. Renal involvement may be overlooked without targeted assessment, including urinalysis, renal function, and electrolyte or acid–base abnormalities (71). Persistent salivary gland swelling, cryoglobulinemia, low complement component 4 (C4) levels, anti-La/SSB positivity, and leukopenia may indicate increased lymphoproliferative risk (72). An abnormal biomarker should therefore prompt assessment of the specific systemic, tissue, or organ-risk domain it is known to represent, rather than automatic treatment escalation.

Biomarker positivity alone should not be assumed to predict treatment response without prospective validation (73, 74). Evidence is strongest for established organ-risk features and organ-specific assessment; biomarker-high/symptom-modest disease remains an interpretive pattern rather than a validated patient classification.

### 5.3 Persistent dysfunction after partial immune improvement: separating biological response, functional recovery, and symptom relief

Persistent dysfunction after partial immune improvement represents a third recurrent pattern of immune–symptom uncoupling. Systemic activity, selected biomarkers, or inflammatory domains may improve, while dryness, glandular dysfunction, pain, fatigue, or patient-reported burden persists. This pattern corresponds to response-layer discordance: evidence of biological response does not ensure parallel functional or symptomatic recovery.

Treatment response can occur across several partially coupled layers. Biological response refers to target engagement or change in pathway-related biomarkers; systemic response refers to change in ESSDAI or organ-domain activity; tissue-level response refers to histopathological or imaging change; objective functional recovery refers to improvement in glandular output, ocular surface status, or organ function; and symptomatic response refers to patient-experienced improvement in dryness, pain, fatigue, or daily functioning. These outcomes are related, but they differ in reversibility, kinetics, and dependence on residual tissue reserve or symptom-processing mechanisms.

Clinically, residual symptoms after immune-targeted therapy should prompt reassessment of both the achieved response and the remaining burden rather than immediate classification as treatment failure. Target engagement and systemic activity should be evaluated separately from tissue or imaging change, objective function, and patient-reported outcomes. Persistent dryness may suggest structural damage, reduced functional reserve, ongoing local disease, or epithelial dysfunction, whereas persistent pain and fatigue may prompt reassessment for neurological involvement, central sensitization or fibromyalgia, sleep disturbance, medication effects, and other comorbid contributors. Evidence for response-layer discordance remains limited because few studies have simultaneously assessed target engagement, tissue change, objective function, and symptoms longitudinally.

## 6 Implications for immune-targeted trials: aligning target, population, endpoint, and timing

For trial design, immune–symptom uncoupling shifts the focus from endpoint choice alone to the alignment of therapeutic target, enrolled population, endpoint selection, and assessment timing (8, 73). Misalignment among these elements may attenuate or obscure domain-specific treatment effects. Four corresponding forms are particularly relevant: target-to-layer, population-to-target, endpoint-to-layer, and timing-window misalignment. **Table 3** summarizes how these forms of misalignment arise and the corresponding design responses.

**Table 3:** Trial-design misalignment and corresponding design responses arising from immune–symptom uncoupling in Sjögren’s disease.

Trial-design misalignment	How it arises	Design response
Target-to-layer	The dominant clinical burden is maintained by structural damage, reduced functional reserve, neurological contributors not addressed by the intervention, symptom amplification, or comorbid conditions; target engagement may therefore not translate into proportional clinical improvement.	Confirm residual target-relevant activity and capacity for change in the corresponding disease layer; pair pharmacodynamic measures with layer-specific outcomes.
Population-to-target	A broad SjD population includes patients with differing pathway activity, organ involvement, residual function, damage burden, and symptom phenotypes; treatment effects may be diluted when the targeted pathway is relevant to only a subset.	Prespecify enrichment or stratification using target-relevant biomarkers or phenotypes, and assess treatment-effect heterogeneity.
Endpoint-to-layer	The primary endpoint captures a layer not expected to change within the trial time frame—for example, symptom burden for a pathway-targeted therapy or systemic activity for a symptom-directed intervention—so a genuine layer-specific response may be missed.	Align the primary endpoint with the expected response layer and report biological, systemic, tissue-level, functional, and patient-reported outcomes separately.
Timing-window	Target engagement, biomarker or systemic change, tissue or functional recovery, and symptomatic improvement may occur on different timescales; a single assessment window may therefore miss the relevant response.	Prespecify separate windows for early pharmacodynamic, intermediate tissue or functional, and later clinical or symptomatic outcomes.

Misalignment types are not mutually exclusive and may coexist within the same trial. The proposed design responses are mechanistically informed principles rather than prospectively validated rules. SjD, Sjögren’s disease.

### 6.1 Target-to-layer and population-to-target misalignment

Target-to-layer misalignment occurs when an intervention modifies one disease layer while the dominant clinical burden in the enrolled population is generated by another. A therapy directed at B-cell activity, IFN signaling, or systemic immune activation may show limited symptomatic benefit when dryness, pain, fatigue, or functional impairment is maintained primarily by structural damage, reduced functional reserve, local tissue pathology, neurological involvement, central sensitization, or comorbid factors.

Population-to-target misalignment is closely related. A diagnostically homogeneous SjD trial cohort may still be biologically heterogeneous, with patients differing in dominant immune pathway, tissue-compartment activity, damage burden, neurological or symptom-amplifying contributors, and organ-risk biology. Evidence from symptom-based stratification and molecular classification studies supports the view that SjD contains clinically and biologically distinct subgroups, but these dimensions only partially overlap (6, 56, 58, 75). A broad trial population may include only a subset of patients in whom the targeted pathway is both active and clinically relevant.

Enrichment strategies should justify the target–outcome link from both directions: why the target is active in the selected patients, and why modifying it should affect the chosen outcome.

The phase 2 dazodalibep trial provides a concrete example of prospective population–endpoint alignment. The study enrolled two distinct populations: participants with moderate-to-severe systemic activity, defined by ESSDAI ≥5, were evaluated using change in ESSDAI as the primary endpoint, whereas participants with ESSPRI ≥5, ESSDAI <5, and residual stimulated salivary flow were evaluated using change in ESSPRI. Both population-specific primary endpoints were met (76). This design illustrates how a single immune-targeted therapy can be evaluated against different dominant clinical domains rather than treating all patients with SjD as one severity population. However, the trial did not directly compare this stratified architecture with an unstratified design and therefore does not establish that stratification itself improved statistical power or predicted individual response. Threshold-defined cohorts may also incompletely represent patients with mixed or intermediate systemic and symptom burden, creating a trade-off between enrichment and external validity across the continuous disease spectrum encountered in routine practice.

### 6.2 Endpoint-to-layer and timing-window misalignment

Endpoint-to-layer misalignment occurs when the primary outcome captures a disease layer that is weakly coupled to the intervention’s expected biological effect. ESSDAI measures systemic organ-domain activity, whereas ESSPRI measures patient-reported dryness, pain, and fatigue (3). Selecting one as the primary endpoint therefore defines the response layer the trial is powered to detect. Importantly, ESSDAI has no dedicated domain for ocular-surface disease or lacrimal function; its glandular domain captures salivary or lacrimal gland swelling rather than tear-film dysfunction or ocular-surface damage (12). Consequently, local ocular treatment effects may not be reflected by changes in systemic activity scores and should be evaluated with domain-matched ocular endpoints. Discordance may also occur within the ocular domain during local treatment. In a randomized trial of autologous-serum and platelet-rich plasma eye drops in primary SjD, corneal and conjunctival staining and tear-film break-up time improved, whereas OSDI scores did not show significant improvement over 12 weeks, illustrating non-parallel objective and symptomatic responses to local ocular therapy (77).

Treatment studies illustrate how responses may differ across endpoint layers. In a phase III trial, abatacept produced changes in disease-relevant immunoglobulins, rheumatoid factor, and immune-cell subsets but did not demonstrate significant clinical efficacy compared with placebo (78). This finding is compatible with divergence between biological activity and measured clinical response, but it does not establish endpoint misalignment as the explanation; insufficient therapeutic efficacy, population heterogeneity, treatment duration, and endpoint sensitivity remain alternative interpretations. In the multicenter rituximab trial, the primary patient-reported endpoint was not met, although unstimulated salivary flow improved; a nested SGUS substudy also demonstrated improvement in total ultrasound scores (79, 80). These findings provide a more direct example of non-parallel symptomatic and glandular responses, although the functional and imaging outcomes were secondary and the SGUS analysis involved a smaller subgroup.

Endpoint selection should match the expected response layer. For therapies directed at B cells or B-cell-activating factor (BAFF), ESSDAI, relevant organ-specific outcomes, and pharmacodynamic measures of B-cell or immunoglobulin activity may be more appropriate primary or major secondary endpoints than ESSPRI alone (8, 73). For IFN-directed therapies, an analytically validated IFN signature may support pathway-based enrichment (81, 82), but systemic or organ-associated outcomes should remain central unless a direct relationship between IFN activity and the selected symptom endpoint has been demonstrated (73).

Glandular or ocular targets may require tissue-informed, imaging-informed, or functional readouts such as SGUS, biopsy-derived features, ocular surface measures, tear production, or salivary flow (83–85). When functional recovery is an expected treatment effect, eligibility criteria should document sufficient residual glandular or ocular function for measurable improvement. Damage-dominant patients should not be enriched for symptom improvement in immune-targeted trials unless residual target-relevant activity is demonstrated. Symptom-directed interventions require patient-reported outcomes, such as ESSPRI or individual dryness, pain, and fatigue measures (3, 86), but these should be interpreted alongside measures that clarify whether improvement reflects immune modulation, functional recovery, or downstream symptom processing.

Timing-window misalignment further complicates interpretation. Immune pathway modulation may occur before changes in glandular function or patient-reported symptoms become measurable, whereas downstream improvement may be constrained by structural damage or reduced functional reserve. Longitudinal cohorts and treatment-response studies show that systemic activity, objective glandular measures, and patient-reported outcomes may follow partially synchronized but non-identical trajectories over time (16, 49). Where feasible, assessment schedules should distinguish early pharmacodynamic target engagement from later changes in tissue activity, function, and symptoms. Trials should specify both the expected response layer and the biologically plausible timing of change.

These endpoint and timing strategies are mechanistically informed design principles rather than prospectively validated rules. A negative primary endpoint may reflect genuine lack of efficacy, insufficient target engagement, inappropriate population selection, endpoint insensitivity, an unsuitable assessment window, or a combination of these factors (73).

### 6.3 Composite endpoints are useful but insufficient without stratified design

Composite endpoints are an important response to multidomain disease expression in SjD (8). The Composite of Relevant Endpoints for Sjögren’s Syndrome (CRESS) and the Sjögren’s Tool for Assessing Response (STAR) were developed to integrate multiple response domains within unified responder frameworks (87, 88). CRESS was developed and retrospectively evaluated using randomized-trial datasets, while STAR was developed through analyses of randomized trials together with expert and patient consensus. Both require further prospective validation as primary trial endpoints.

Composite endpoints address only part of the problem. An overall composite response may be driven by improvement in only one or a subset of components, while other clinically important layers remain unchanged. Composite responder rates should therefore be accompanied by component-level results and, where appropriate, sensitivity analyses identifying which components account for the observed treatment effect.

Composite endpoints should complement rather than replace separate reporting of biological, systemic, tissue-level, functional, and patient-reported outcomes. Current evidence supports the feasibility and potential responsiveness of CRESS and STAR, but does not establish their universal superiority over single-domain endpoints or their ability to resolve inappropriate population selection and treatment–target misalignment.

## 7 Future directions

Immune–symptom uncoupling provides a conceptual framework for interpreting discordance in SjD, but its clinical and trial-design utility requires prospective validation. Future longitudinal, multimodal, and treatment-linked studies should test when, why, and in whom specific disease domains diverge.

### 7.1 Mapping longitudinal discordance trajectories

A key unresolved question is how discordance changes over time. Cross-sectional assessment can identify discordance at a single visit, but cannot determine whether it reflects stable symptom-high/activity-low disease, delayed downstream recovery, evolving systemic activity, progressive glandular dysfunction, or persistent damage-dominant burden. For this reason, divergence between domains should be prespecified and analyzed as an outcome in its own right, not merely as inconsistency among separate measures.

Existing longitudinal studies have shown that glandular function, systemic activity, and patient-reported outcomes may follow non-identical trajectories over time (16, 47). Building on this work, future cohorts should use repeated, synchronized assessments of systemic activity, circulating biomarkers, tissue or imaging findings, objective glandular and ocular function, and patient-reported outcomes, while recording treatment exposure and major symptom-modifying factors. Such designs may clarify whether symptom-high/activity-low or biomarker-high/symptom-modest presentations are reproducible disease states, dynamic phases, or consequences of non-synchronized measurement windows, and whether changes in one layer precede or follow changes in another. Discordance can then be analyzed as a longitudinal variable, rather than dismissed as a cross-sectional inconsistency. At present, few cohorts have repeatedly assessed all relevant layers within the same patients, leaving the stability and temporal ordering of discordance incompletely defined.

### 7.2 Developing compartment-aware and layer-specific biomarkers

Another challenge is to develop biomarkers that are explicitly linked to the disease layer they measure. Blood-based biomarkers remain essential for identifying systemic immune activation, but they cannot fully represent glandular inflammation, epithelial dysfunction, stromal remodeling, fibrosis, imaging abnormalities, functional reserve, or neurological and symptom-amplifying processes. The key question is what biological layer a biomarker reports, what outcome it is expected to predict, and whether these relationships extend beyond correlations with global disease severity.

Histopathology, salivary flow, and SGUS capture complementary aspects of glandular involvement (13), while epithelial–immune crosstalk, spatial immune niches, and autoantibody-associated tissue programs suggest that local pathology may require tissue-informed or imaging-informed readouts (23, 24, 61). Integrating serology, genomic, transcriptomic, proteomic, or metabolomic data, imaging, biopsy-derived features, functional measures, and patient-reported outcomes may help identify layer-specific biomarker profiles. Such integration should preserve the distinction between systemic immune activation, local tissue pathology, objective function, and patient-experienced burden rather than combining all measurements into a single severity score.

Machine learning and other computational approaches may support multimodal integration, clustering, trajectory modeling, and treatment-response prediction, while causal-inference analyses require appropriately designed longitudinal or treatment-linked data. In SjD, multi-omic analysis of molecular, cellular, and clinical data has identified candidate biological subgroups and generated classifiers for subgroup assignment (52). Unsupervised integration of symptoms with clinical and biological variables has also identified B-cell-active/low-symptom, high-systemic-activity, and low-systemic-activity/high-symptom subgroups that were reproduced in an independent cohort and showed different longitudinal outcomes (1). Digital pathology studies further demonstrate the feasibility of automated glandular classification and tissue-based risk prediction, but have not yet established identification of immune–symptom uncoupling or differential treatment response (89, 90). These applications remain exploratory because small, selected, and high-dimensional datasets create risks of overfitting and unstable subgroup assignment.

Future studies should define the intended prediction task in advance and use longitudinal data to test whether multimodal models distinguish stable discordance from temporal asynchrony or improve prediction of organ progression and treatment response. Models should be externally validated, compared with simpler clinical approaches, and assessed for calibration, interpretability, reproducibility, handling of missing modalities, and subgroup stability. Computational complexity alone should not be treated as evidence of clinical utility.

### 7.3 Testing layer-informed enrichment, response prediction, and clinical utility

The clinical value of this framework will ultimately depend on whether layer-informed stratification improves prediction of treatment response. Endotype-informed trials should enroll patients with evidence that the targeted pathway is active, clinically relevant, and aligned with the outcome expected to change (52, 73, 74). For immune-targeted therapies, enrichment may require molecular, serological, laboratory, imaging, or tissue-compartment evidence that the targeted pathway is active, not symptom burden alone. Candidate stratification rules should be defined before outcome analysis and subsequently evaluated in independent populations, rather than derived and tested within the same dataset.

Future studies should also distinguish response layers prospectively. Biological target engagement, systemic activity improvement, tissue or imaging change, glandular or ocular functional recovery, and patient-reported symptom relief should be analyzed as related but non-interchangeable outcomes. This distinction would help separate biological failure from biological success without functional recovery, and from symptomatic improvement mediated outside systemic immune modulation. The critical test of a proposed subgroup is whether it identifies reproducible treatment-effect heterogeneity—that is, whether patients assigned prospectively to that subgroup respond differently to a biologically matched intervention. Such designs would move SjD trials from broad diagnostic enrollment toward mechanism-aligned patient selection, endpoint choice, and response interpretation.

### 7.4 Evidence limitations and validation requirements

The current evidence base also has important limitations. Much of the evidence supporting immune–symptom uncoupling comes from cross-sectional cohorts, observational associations, *post hoc* analyses, or studies designed for other primary questions. Tissue-compartment, single-cell, and spatial studies are frequently limited by small and selected samples, while symptom and neurological studies use heterogeneous definitions and measurement methods. Differences in classification criteria, outcome instruments, follow-up intervals, and adjustment for fibromyalgia, sleep disturbance, depression, autonomic dysfunction, medication effects, and non-SjD causes of symptoms further limit comparison across studies. Selection and publication bias are also difficult to exclude. These data support a biologically plausible framework but do not fully establish causal relationships among immune activity, tissue pathology, neurological or symptom-amplifying processes, and patient burden.

Before routine clinical or trial use, layer-informed classifications should demonstrate reproducibility, appropriate longitudinal stability, external validity, and incremental value beyond simpler clinical measures. Treatment-predictive claims require prospective confirmation that a classification modifies response to a specific intervention, rather than merely being associated with prognosis or baseline disease severity. The priority is therefore to determine which layer-specific phenotypes are reproducible, clinically actionable, and predictive of treatment response, rather than to generate additional subgroups for their own sake. In this sense, immune–symptom uncoupling remains a testable framework, not a settled explanatory model.

## 8 Conclusion

Immune–symptom uncoupling is a recurrent feature of SjD expression. Across systemic activity–symptom discordance, tissue–function discordance, biomarker–phenotype discordance, and response-layer discordance, incomplete alignment should be understood as partial, context-dependent, and sometimes temporally variable rather than as complete biological separation. Symptoms are not direct readouts of systemic immune activity; their relationship with immune activity may be modified by local tissue pathology, active functional or bioenergetic dysregulation, structural injury and reduced functional reserve, SjD-related neurological involvement, central sensitization and symptom amplification, comorbid or contextual modifiers, and temporal asynchrony. This helps explain why high symptom burden may coexist with limited systemic activity, why biomarker- or endotype-defined disease may occur with modest symptoms, and why glandular dysfunction or patient-reported burden may persist after partial immune improvement.

A mechanism-informed interpretation of discordance requires asking which disease layer is active, which layer is being measured, and which layer is expected to change. For clinical care, this approach supports targeted assessment of the dominant modifiable contributor rather than automatic escalation of immunosuppression or dismissal of symptoms. For biomarker research and immune-targeted trials, it emphasizes the need to align markers, patient selection, endpoints, and assessment windows with the biological process of interest. Future longitudinal and multimodal studies should test whether prospectively defined layer-specific classifications add reproducible prognostic or treatment-predictive value beyond simpler clinical measures. Until such validation is available, immune–symptom uncoupling should guide multidomain interpretation rather than be used to assign a single cause to an individual patient’s symptoms.
